# Antibody Kinetics and Response to Routine Vaccinations in Infants Born to Women Who Received an Investigational Trivalent Group B *Streptococcus* Polysaccharide CRM_197_-Conjugate Vaccine During Pregnancy

**DOI:** 10.1093/cid/cix666

**Published:** 2017-09-23

**Authors:** Shabir A Madhi, Anthonet Koen, Clare L Cutland, Lisa Jose, Niresha Govender, Frederick Wittke, Morounfolu Olugbosi, Ajoke Sobanjo-ter Meulen, Sherryl Baker, Peter M Dull, Vas Narasimhan, Karen Slobod

**Affiliations:** 1 Medical Research Council: Respiratory and Meningeal Pathogens Research Unit; 2 Department of Science and Technology/National Research Foundation: Vaccine Preventable Diseases, University of the Witwatersrand; 3 National Institute for Communicable Diseases, National Health Laboratory Service, Centre for Vaccines and Immunology, Johannesburg, South Africa; 4 GSK and Novartis Vaccines Division, Cambridge, Massachusetts

**Keywords:** antenatal vaccination, group B *Streptococcus* conjugate vaccine, routine immunization, infants

## Abstract

**Background:**

Maternal vaccination against group B *Streptococcus* (GBS) might provide protection against invasive GBS disease in infants. We investigated the kinetics of transplacentally transferred GBS serotype-specific capsular antibodies in the infants and their immune response to diphtheria toxoid and pneumococcal vaccination.

**Methods:**

This phase 1b/2, observer-blind, single-center study (NCT01193920) enrolled infants born to women previously randomized (1:1:1:1) to receive either GBS vaccine at dosages of 0.5, 2.5, or 5.0 μg of each of 3 CRM_197_-glycoconjugates (serotypes Ia, Ib, and III), or placebo. Infants received routine immunization: combination diphtheria vaccine (diphtheria-tetanus-acellular pertussis–inactivated poliovirus/*Haemophilus influenzae* type b vaccine; age 6/10/ 14 weeks) and 13-valent pneumococcal CRM_197_-conjugate vaccine (PCV13; age 6/14 weeks and 9 months). Antibody levels were assessed at birth, day (D) 43, and D91 for GBS serotypes; 1 month postdose 3 (D127) for diphtheria; and 1 month postprimary (D127) and postbooster (D301) doses for pneumococcal serotypes.

**Results:**

Of 317 infants enrolled, 295 completed the study. In infants of GBS vaccine recipients, GBS serotype-specific antibody geometric mean concentrations were significantly higher than in the placebo group at all timepoints and predictably decreased to 41%–61% and 26%–76% of birth levels by D43 and D91, respectively. Across all groups, ≥95% of infants were seroprotected against diphtheria at D127 and ≥91% of infants had seroprotective antibody levels against each PCV13 pneumococcal serotype at D301.

**Conclusions:**

Maternal vaccination with an investigational CRM_197_-glycoconjugate GBS vaccine elicited higher GBS serotype-specific antibody levels in infants until 90 days of age, compared with a placebo group, and did not affect infant immune responses to diphtheria toxoid and pneumococcal vaccination.

**Clinical Trials Registration:**

NCT01193920.

## Teaser

Group B *Streptococcus* (GBS)–specific antibodies were detectable in 3-month-old infants of mothers having received an investigational trivalent GBS vaccine during pregnancy. Maternal GBS vaccination did not have a clinically meaningful impact on immune responses to diphtheria or pneumococcal pediatric vaccination.

Group B *Streptococcus* (GBS) is a prominent cause of morbidity in neonates worldwide [1], and also causes invasive disease with a higher incidence in pregnant women than in nonpregnant adults [2]. The burden of invasive GBS disease is especially high in infants from low- and middle-income countries [1, 3–5], with the disease presenting as early-onset (in the first 7 days of life) or late-onset (from 8 days through 3 months). Implementation of intrapartum antibiotic prophylaxis reduced early-onset neonatal disease [6, 7] but had little impact on the incidence of late-onset invasive GBS disease [6, 8]. The development of a vaccine to be administered during pregnancy could be a more suitable strategy for the prevention of invasive GBS disease in infants throughout the first 3 months of life, the period with the highest likelihood of GBS invasive disease onset [6]. High maternal GBS serotype-specific capsular antibody levels have been correlated with protection of infants against early-onset disease [9–11], but the extension of this protection beyond the first week of life remains a need that maternal vaccination might meet [12].

Maternal antibodies have, however, been reported to interfere with immune responses during active immunization of infants [13]. An attenuated immune response to diphtheria, pertussis, and some pneumococcal vaccine serotypes (conjugated to diphtheria-toxin mutant carrier protein [CRM_197_]) was observed in infants born to mothers who had received an acellular pertussis-diphtheria-tetanus toxoid (TT) vaccine during pregnancy, compared with a historical cohort of infants [14].

Although no vaccine is currently licensed for use against GBS, some capsular polysaccharide-protein conjugate candidate vaccines using either TT or CRM_197_ as carrier proteins have reached phase 2 [12]. Several formulations and dosages of an investigational trivalent vaccine containing capsular polysaccharides of GBS serotypes Ia, Ib and III, each individually conjugated to CRM_197_, were shown to induce a persistent antibody response in healthy nonpregnant women [15].

We have previously reported the safety and immunogenicity of an adjuvanted formulation of the investigational GBS vaccine in nonpregnant women, and of a nonadjuvanted investigational vaccine dose containing different dosages of GBS glycoconjugates in pregnant women and their infants [16]. Higher GBS serotype-specific antibody concentrations were observed at delivery in infants born to mothers who had received the GBS vaccine compared with the placebo during pregnancy, and all vaccine formulations and dosages were well tolerated [16]. In this article, we describe the kinetics of GBS-specific antibodies during the first 3 months of life among infants born to women immunized during pregnancy with the CRM_197_-conjugate GBS vaccine. The impact of maternal GBS vaccination on infant diphtheria and pneumococcal antibody levels following routine immunization in the first year of life is also evaluated.

## METHODS

### Study Design and Participants

This phase 1b/2 randomized, observer-blind clinical study was conducted between October 2010 and December 2012 in Soweto, South Africa. The trial complied with the International Council for Harmonisation of Technical Requirements for Pharmaceuticals for Human Use (ICH) Harmonised Tripartite Guidelines for Good Clinical Practice and the Declaration of Helsinki. Written informed consent was obtained from a parent/guardian of all infants prior to enrolment, and the protocol and amendments were approved by the Human Research Ethics Committee of the University of the Witwatersrand and the Medicines Control Council (South Africa). The study was registered at ClinicalTrials.gov (NCT01193920).

The study design and outcomes related to primary objectives have been described in detail [16]. Herein we present the results of secondary objectives, assessed in infants born at least 2 weeks after vaccination to human immunodeficiency virus–negative, pregnant women enrolled in the study, who had received the investigational vaccine correctly, at 28–35 weeks of gestation. Infants were enrolled within 4 days from birth from healthy women previously randomized in a 1:1:1:1 ratio to receive a single injection of 1 of 3 dosages of the investigational GBS vaccine (0.5 μg, 2.5 μg, or 5.0 μg of each of 3 CRM_197_-capsular glycoconjugates representing GBS serotypes Ia, Ib and III, with a CRM_197_ content of 0.6–3 μg, 3–15 μg, and 6–30 μg, respectively, or placebo (0.9% sodium chloride).

As part of the routine immunization program, infants received a diphtheria-tetanus-acellular pertussis–inactivated poliovirus/*Haemophilus influenzae* type b vaccine (DTaP-IPV/Hib; Pentaxim, Sanofi Pasteur) at 6, 10, and 14 weeks of age and a 13-valent pneumococcal conjugate vaccine (PCV13; Prevenar 13, Pfizer) at 6 weeks, 14 weeks, and 9 months of age. Both routine vaccines (0.5 mL doses) were administered intramuscularly into the anterolateral thigh.

### Immunogenicity Assessments

Immunogenicity analyses were carried out in the per-protocol set, including infants who complied with the eligibility criteria and had evaluable serum samples at the protocol-defined time points. The GBS antibody levels against the 3 vaccine serotypes were evaluated at birth (day [D] 0), D43, and D91. Immune response to diphtheria was evaluated at D127 (1 month following the third DTaP-IPV/Hib dose), and to each PCV13 serotype at D127 (1 month postprimary vaccination) and D301 (1 month postbooster).

Blood samples (0.5 mL) collected from infants were analyzed at GSK, Marburg, Germany (GBS), Rochester General Research Institute Laboratory, New York (diphtheria), or University College London, United Kingdom (PCV13). The enzyme-linked immunosorbent assay (ELISA) used for estimating GBS-specific antibody concentrations has been described previously [17]. The lower limits of quantitation (LLQs) were 0.326 μg/mL, 0.083 μg/mL, and 0.080 μg/mL for GBS serotypes Ia, Ib, and III, respectively. Diphtheria and serotype-specific pneumococcal antibody concentrations were determined by ELISA using existing protocols [18, 19].

### Statistical Analysis

Adjusted GBS serotype-specific antibody geometric mean concentrations (GMCs) were calculated with associated 2-sided 95% confidence intervals (CIs). Antibody concentrations below LLQ were given an arbitrary unit of half the LLQ for GMC calculations. GBS antibody half-life estimation and exploratory analyses are described in the Supplementary Materials. The null hypothesis of no difference between 2 dosage groups was tested at a significance level of .05 with no multiplicity adjustments. Responses in 2 dosage groups were concluded to be different if there was a significant difference for at least 2 of 3 serotypes.

Immune responses to diphtheria and PCV13 serotypes were presented as the proportion of infants with concentrations above the putative correlates of protection of 0.1 IU/mL and 0.35 µg/mL, respectively, in agreement with serological criteria defined by the World Health Organization for evaluation of diphtheria [20] and pneumococcal [21] vaccines, together with unadjusted antibody GMCs and corresponding 95% CIs.

All 95% CIs were calculated using the Clopper-Pearson method. Statistical analyses were performed using SAS 9.1 software (SAS Institute, Cary, North Carolina).

## RESULTS

### Study Population

In total, 317 infants born to 315 pregnant women (98% of the total vaccinated cohort) were enrolled, of whom 295 (93%) completed the study. Reasons for withdrawal were death or stillbirth (n = 11), loss to follow-up (n = 6), withdrawal of consent (n = 4), and relocation (n = 1). Demographics of infants and GBS serotype-specific antibody transfer ratios from mother to infant, measured at delivery, have previously been published [16].

### Persistence of Immune Response to Group B *Streptococcus* in Infants Following Maternal Vaccination

In all GBS groups, infant GBS serotype-specific antibody GMCs were significantly higher than in the placebo group across all timepoints (Table 1). At D43, in GBS groups, GBS antibody GMCs were within the 2.95–5.54 μg/mL range for serotype Ia, 0.67–0.88 μg/mL range for serotype Ib, and 0.68–0.88 μg/mL range for serotype III, significantly higher than in the placebo group (0.31 μg/mL, 0.15 μg/mL, and 0.16 μg/mL for serotypes Ia, Ib, and III, respectively). By D91, GBS antibody GMCs in GBS groups ranged from 1.97 to 2.78 μg/mL for serotype Ia, from 0.83 to 1.08 μg/mL for serotype Ib, and from 0.51 to 0.69 μg/mL for serotype III, compared with 0.38 μg/mL, 0.50 μg/mL, and 0.27 μg/mL, respectively, in the placebo group (Table 1).

**Table 1.** Summary of Immune Responses Against Group B *Streptococcus* Serotypes

Serotype	Day	Placebo^a^		GBS 0.5 μg		GBS 2.5 μg		GBS 5.0 μg	
		No.	Value (95% CI)	No.	Value (95% CI)	No.	Value (95% CI)	No.	Value (95% CI)
GBS serotype Ia									
GMC	Birth	73	0.49 (.34–.71)	75	6.66 (4.03–11.0)	76	6.52 (3.96–11.0)	69	12.0 (7.21–20.0)
GMC	D43	71	0.31 (.23–.42)	75	3.21 (2.01–5.13)	76	2.95 (1.85–4.70)	68	5.54 (3.38–9.06)
GMC	D91	75	0.38 (.29–.49)	71	1.97 (1.31–2.95)	75	2.22 (1.49–3.29)	64	2.78 (1.81–4.26)
Ratio	D43:Birth	68	0.67 (.58–.77)	74	0.51 (.44–.58)	75	0.47 (.41–.54)	68	0.45 (.39–.52)
Ratio	D91:Birth	71	0.81 (.63–1.05)	70	0.31 (.25–.39)	73	0.32 (.25–.40)	63	0.26 (.20–.33)
GBS serotype Ib									
GMC	Birth	62	0.21 (.14–.32)	71	1.15 (.68–1.95)	71	1.72 (1.02–2.92)	66	1.39 (.81–2.40)
GMC	D43	69	0.15 (.11–.21)	72	0.73 (.47–1.14)	77	0.88 (.57–1.35)	67	0.67 (.42–1.07)
GMC	D91	76	0.50 (.39–.63)	74	0.85 (.61–1.18)	73	1.08 (.77–1.51)	66	0.83 (.59–1.19)
Ratio	D43:Birth	57	0.82 (.62–1.08)	69	0.61 (.51–.73)	70	0.59 (.49–.71)	64	0.52 (.43–.63)
Ratio	D91:Birth	61	2.40 (1.68–3.43)	69	0.76 (.53–1.08)	67	0.69 (.49–.99)	62	0.66 (.45–.95)
GBS serotype III^b^									
GMC	Birth	52	0.29 (.19–.43)	57	2.11 (1.26–3.54)	64	2.30 (1.41–3.76)	58	1.72 (1.03–2.87)
GMC	D43	63	0.16 (.12–.22)	64	0.82 (.53–1.27)	75	0.88 (.59–1.31)	61	0.68 (.43–1.06)
GMC	D91	69	0.27 (.21–.34)	63	0.60 (.42–.85)	63	0.69 (.48–.98)	57	0.51 (.35–.74)
Ratio	D43:Birth	44	0.57 (.44–.73)	48	0.49 (.39–.62)	62	0.41 (.34–.50)	51	0.44 (.36–.55)
Ratio	D91:Birth	46	0.83 (.60–1.14)	45	0.35 (.24–.49)	53	0.29 (.21–.39)	47	0.32 (.23–.45)

Data are shown as antibody GMCs and GMC ratios at days 43 and 91 compared with baseline, by timepoint (per-protocol set).

Abbreviations: CI, confidence interval; D, day; GBS, group B *Streptococcus*; GMC, geometric mean concentration; No., number of infants with available results in each group.

^a^GBS-specific antibody geometric mean concentrations at birth were negligible in the placebo group and therefore ratios for this group are unreliable.

^b^GBS-specific antibody concentrations were assessed by priority ranking, following the order Ia > Ib > III. Due to limitations in serum volume, it was not possible for all sera to generate final results in all 3 serotype enzyme-linked immunosorbent assays.

Infant GBS antibody GMCs for serotypes Ia and III decreased to 41%–51% of levels at birth by D43 and to 26%–35% by D91, across all GBS groups. While GMC ratios suggested a decrease in antibody GMCs for serotype Ib at D43 (52%–61%) and D91 (66%–76%) from values at birth in the GBS-vaccinated groups, GMC values had overlapping CIs at all timepoints (Table 1). The estimated GBS serotype-specific antibody half-life ranged from 39 to 46 days for the 3 serotypes.

Exploratory analyses showed no substantial differences in GBS serotype-specific antibody levels among infants in GBS groups born from mothers vaccinated at 28 to <30, 30 to <32, 32 to <34, or ≥34 weeks of gestation (Supplementary Table 1). Further exploratory analyses showed that, among infants born to mothers with GBS antibody GMCs below the LLQ at baseline, infants in the GBS groups showed higher antibody concentration at birth, D43, and D91 than infants in the placebo group (Table 2). Across all GBS groups, the persistence of GBS serotype-specific antibody levels from birth up to 3 months of age was lower in infants born to mothers with antibody levels below LLQ at prevaccination than in the overall infant population (Tables 1–2).

**Table 2.** Group B *Streptococcus* (GBS) Serotype-Specific Antibody Geometric Mean Concentrations (GMCs) in Infants Born to Mothers With Prevaccination GBS Antibody GMCs Below the Lower Limit of Quantitation, by Timepoint (Per-Protocol Set)

Serotype	Placebo	GBS 0.5 μg	GBS 2.5 μg	GBS 5.0 μg
	GMC (95% CI)	GMC (95% CI)	GMC (95% CI)	GMC (95% CI)
GBS serotype Ia	No. = 31	No. = 20	No. = 26	No. = 24
Birth	0.18 (.15–.22)	1.43 (.58–3.53)	3.14 (1.42–6.92)	3.18 (1.40–7.25)
D43	0.18 (.15–.21)	0.80 (.34–1.84)	1.32 (.63–2.76)	1.59 (.74–3.42)
D91	0.27 (.20–.37)	0.74 (.37–1.48)	1.01 (.55–1.87)	0.86 (.45–1.62)
GBS serotype Ib	No. = 11	No. = 9	No. = 11	No. = 8
Birth	0.09 (.06–.14)	0.54 (.14–2.10)	0.76 (.22–2.62)	0.23 (.05–.97)
D43	0.08 (.05–.13)	0.29 (.10–.90)	0.55 (.20–1.53)	0.15 (.05–.50)
D91	0.23 (.11–.47)	0.49 (.18–1.33)	0.51 (.21–1.25)	0.44 (.15–1.28)
GBS serotype III	No. = 3	No. = 3	No. = 4	No. = 4
Birth	0.07 (.02–.27)	0.33 (.04–2.89)	0.85 (.13–5.56)	0.29 (.04–1.91)
D43	0.07 (.01–.69)	0.40 (.09–1.90)	0.40 (.10–1.52)	0.17 (.05–.66)
D91	0.23 (.02–2.16)	0.51 (.16–1.59)	0.41 (.15–1.10)	0.29 (.11–.78)

Abbreviations: CI, confidence interval; D, day; GBS, group B *Streptococcus*; GMC, geometric mean concentration; No., number of infants with available results in each group.

### Immune Response to Diphtheria and Pneumococcal Vaccination

**Figure 1.**
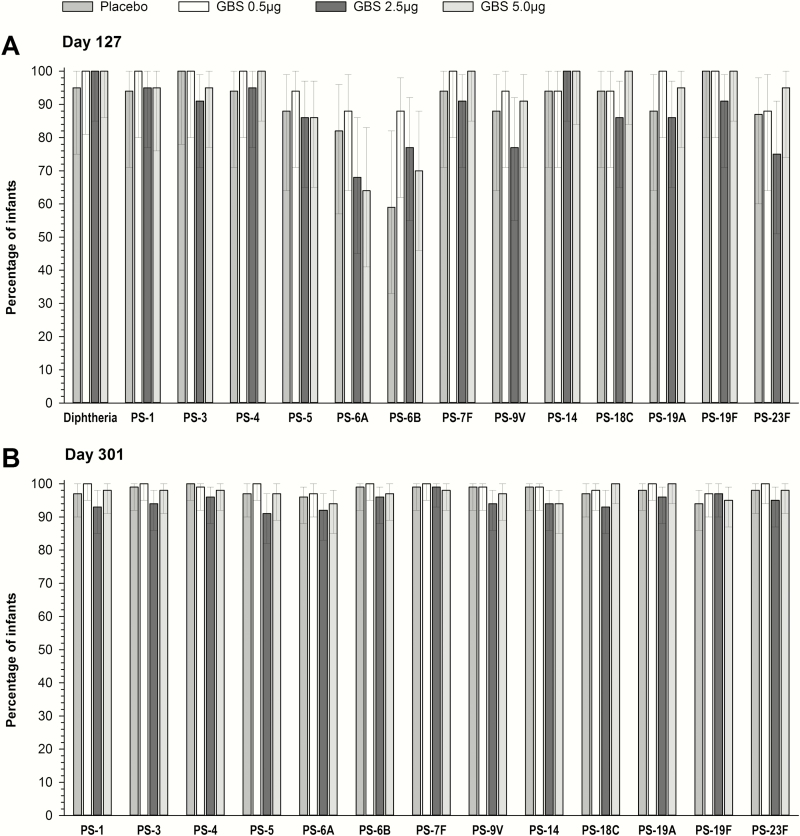
Percentage of infants with enzyme-linked immunosorbent assay antibody concentrations ≥0.1 IU/mL against diphtheria (day 127) and ≥0.35 μg/mL against pneumococcal serotypes (PS) (days 127 [*A*] and 301 [*B*]), by timepoint (per-protocol set). Error bars indicate 95% confidence intervals. Abbreviation: GBS, Group B Streptococcus.

All infants in the GBS groups and 95% of infants in the placebo group achieved diphtheria antibody concentrations ≥0.1 IU/mL after DTaP-IPV/Hib vaccination (Figure 1). Diphtheria antibody GMCs at D127 across all groups ranged from 1.49 to 2.43 IU/mL (Table 3).

**Table 3.** Antidiphtheria and Antipneumococcal Antibody Geometric Mean Concentrations, by Timepoint (Per-Protocol Set)

		Placebo		GBS 0.5 μg		GBS 2.5 μg		GBS 5.0 μg	
		No.^a^	GMC (95% CI)	No.^a^	GMC (95% CI)	No.^a^	GMC (95% CI)	No.^a^	GMC (95% CI)
Diphtheria	D127	20	2.40 (1.64–3.51)	18	2.43 (1.63–3.63)	22	1.74 (1.21–2.50)	24	1.49 (1.05–2.10)
PS-1	D127	17	3.69 (2.04–6.66)	17	4.24 (2.35–7.67)	22	3.64 (2.16–6.13)	21	3.45 (2.03–5.88)
	D301	69	6.97 (5.32–9.14)	67	7.74 (5.89–10.0)	72	6.03 (4.63–7.86)	63	6.08 (4.58–8.06)
PS-3	D127	15	1.78 (1.11–2.86)	17	1.98 (1.27–3.09)	22	1.62 (1.10–2.39)	22	1.88 (1.27–2.77)
	D301	67	2.45 (1.97–3.04)	66	2.58 (2.08–3.22)	69	2.28 (1.84–2.83)	62	2.37 (1.89–2.96)
PS-4	D127	17	2.86 (1.71–4.78)	17	3.28 (1.96–5.48)	22	2.85 (1.81–4.47)	22	2.81 (1.79–4.41)
	D301	69	4.54 (3.60–5.71)	67	4.76 (3.77–6.02)	72	3.73 (2.97–4.67)	64	3.58 (2.81–4.54)
PS-5	D127	17	1.44 (.82–2.51)	17	1.32 (.76–2.31)	22	1.26 (.77–2.05)	22	1.29 (.79–2.11)
	D301	69	2.53 (2.00–3.20)	67	2.94 (2.32–3.73)	70	2.06 (1.63–2.59)	65	2.12 (1.67–2.70)
PS-6A	D127	17	1.48 (.64–3.42)	17	2.36 (1.03–5.44)	22	1.54 (.74–3.20)	22	1.13 (.54–2.36)
	D301	68	8.20 (5.72–12.0)	68	10.0 (6.97–14.0)	72	5.91 (4.16–8.39)	63	6.99 (4.81–10.0)
PS-6B	D127	17	0.82 (.35–1.92)	16	1.68 (.70–4.03)	22	1.32 (.62–2.78)	20	0.86 (.40–1.89)
	D301	68	11.0 (8.56–15.0)	68	8.81 (6.60–12.0)	72	7.74 (5.85–10.0)	62	8.69 (6.42–12.0)
PS-7F	D127	17	4.34 (2.33–8.09)	17	5.44 (2.92–10.0)	22	4.41 (2.55–7.63)	22	4.72 (2.73–8.16)
	D301	68	9.36 (7.51–12.0)	68	12.0 (9.90–15.0)	72	9.06 (7.32–11.0)	64	8.40 (6.70–11.0)
PS-9V	D127	17	2.11 (1.13–3.92)	17	2.28 (1.23–4.24)	22	1.39 (.81–2.40)	22	2.05 (1.19–3.53)
	D301	68	4.79 (3.79–6.06)	68	4.94 (3.90–6.25)	72	3.50 (2.78–4.39)	63	4.09 (3.20–5.22)
PS-14	D127	17	3.34 (1.76–6.33)	17	3.86 (2.04–7.32)	22	4.41 (2.51–7.74)	21	4.28 (2.41–7.61)
	D301	67	13.0 (9.30–18.0)	68	9.82 (7.09–14.0)	71	6.28 (4.56–8.64)	63	6.09 (4.34–8.55)
PS-18C	D127	17	2.78 (1.53–5.03)	17	2.50 (1.38–4.53)	22	2.10 (1.25–3.54)	21	2.30 (1.35–3.93)
	D301	69	5.75 (4.36–7.60)	66	6.67 (5.02–8.87)	72	4.74 (3.61–6.23)	64	5.52 (4.13–7.37)
PS-19A	D127	17	1.54 (.75–3.15)	17	2.92 (1.43–5.96)	22	2.60 (1.39–4.87)	22	3.42 (1.83–6.41)
	D301	65	8.57 (6.55–11.0)	68	8.80 (6.76–11.0)	71	5.62 (4.34–7.27)	63	8.78 (6.68–12.0)
PS-19F	D127	17	5.14 (2.66–9.92)	17	3.64 (1.88–7.02)	22	6.86 (3.85–12.0)	22	5.78 (3.24–10.0)
	D301	68	7.92 (5.83–11.0)	68	7.74 (5.70–11.0)	72	6.69 (4.97–9.01)	64	8.90 (6.49–12.0)
PS-23F	D127	15	2.00 (.90–4.45)	17	2.87 (1.35–6.10)	20	2.25 (1.12–4.50)	19	3.51 (1.72–7.15)
	D301	63	13.0 (10.0–18.0)	64	12.0 (8.72–15.0)	66	8.28 (6.25–11.0)	60	10.0 (7.63–14.0)

Abbreviations: CI, confidence interval; D, day; GBS, group B *Streptococcus*; GMC, geometric mean concentration; No., number of infants with available results in each group; PS, pneumococcal serotype.

^a^The number of infants evaluated at D127 is lower than at D301 due to the fact that the assessment of immune responses to diphtheria and pneumococcal serotypes was included in an amended study protocol at a time when the majority of children had already completed the D127 visit. Therefore, blood draw was only performed for the remaining 36% of enrolled infants for this timepoint.

Between 59%–100% and 91%–100% of infants in all groups had antibody concentrations ≥0.35 μg/mL against each vaccine pneumococcal serotype following primary and booster PCV13 vaccination, respectively (Figure 1), and there were no statistically significant differences between groups. One month post–booster dose (D301), significant increases in antibody GMCs were observed for serotypes 6A, 6B, 18C, 19A, and 23F compared to postprimary vaccination (D127) levels (Table 3). Antibody levels were similar between the placebo and the GBS groups for all serotypes, except serotype 14, for which lower antibody GMCs were observed after the PCV13 booster dose in the GBS 2.5 μg and 5.0 μg groups compared to the placebo group (Table 3).

## DISCUSSION

Maternal GBS immunization is intended to protect infants throughout the period of vulnerability, stretching from birth to 3 months of age. This study evaluated for the first time the immune responses induced in infants born to women having received 1 of 3 different dosages of an investigational trivalent glycoconjugate vaccine against GBS. Regardless of dosage, lower GMCs were observed for serotypes Ib and III than for Ia, similarly with previous observations following administration of the GBS trivalent vaccine in the infants’ mothers or nonpregnant women [15, 16]. Following vaccination, maternal antibody transfer rates at birth ranged from 49% to 79% across the 3 investigational vaccine dosages and across the 3 GBS serotypes [16]. At D43, infant antibody GMCs for all investigational vaccine serotypes ranged from 41% to 61% of the levels measured at birth. Infants in our study still had a large proportion of the antibody levels measured at birth around the median age of late-onset disease, which varies from 14 to 33 days of age, depending on geographic and economic settings [3, 22–26]. Antibody levels of 26%–76% of values at birth were still detected at D91, indicating that placentally transferred GBS-specific antibodies were present throughout the entire period of infant vulnerability to GBS-invasive disease, as around 70% of GBS cases with late onset were reported to occur during the first 2 months of age [27].

The data for the GBS 5.0 μg group are in line with previously reported results in infants born from European and Canadian women receiving the same formulation [17]. A previous study assessing the immunogenicity of an investigational monovalent GBS vaccine also reported antibody persistence levels similar to those seen in our study, with 30% of initial infant serotype III–specific antibody concentrations present 60 days after birth [28]. However, the clinical relevance of these results is still unclear [29]. In recent studies using Bayesian modeling, a maternal serum serotype-specific antibody concentration of ≥1 μg/mL at the time of delivery was proposed as correlate of protection against early-onset GBS disease caused by serotypes Ia and III in North American infants [9]. In South Africa, a setting with a high burden of GBS invasive disease, more stringent thresholds of maternal antibody concentrations at time of delivery of ≥6 μg/mL and ≥3 μg/mL were estimated for prevention of early- or late-onset serotypes Ia and III invasive disease, with a transplacental antibody transfer ratio to healthy newborns of 0.67–0.90 [30]. The difference between the thresholds proposed in these 2 studies may also be explained by the time at which the samples were collected (at delivery [9] or at 3–4 days from birth [30]), with South African mothers having a longer period of time to potentially mount a response to GBS. Another potential explanation is that different analytical methods were used to determine antibody concentrations. Additional data are needed to accurately estimate the protective thresholds and their evolution over the first 3 months of life, as the infant’s immune system starts maturing during that time. Furthermore, direct comparison of antibody levels needs to be interpreted with caution in the absence of a standardized antibody assay, despite the same reference sera being used in all these studies.

The timing of the investigational vaccine administration during pregnancy was previously reported to affect antibody levels and persistence in infants for pertussis maternal vaccination [31, 32]. However, in our study, the persistence of GBS antibody levels in infants did not seem to be impacted by the gestational age at vaccination. Further subgroup analysis indicated that antibody concentrations in infants born to women with prevaccination levels below the LLQ were lower than in the overall population throughout the 3 first months of life, similar to observations reported in the vaccinated pregnant women [16]. Still, these concentrations were higher in the GBS groups than in the placebo group, indicating that the infants might benefit from the vaccine-induced response in their mothers.

The half-life of GBS serotype-specific antibodies ranged between 39 and 46 days, which is in line with the half-life of other maternal transferred antibodies (47 days for pertussis [32], 40 days for hepatitis A [33], 43–45 days for influenza virus antibodies [34], and 38 days for respiratory syncytial virus [35]), and longer than the reported half-life of IgG following intravenous administration of immunoglobulin in neonates (24.2 days) [36].

The importance of maternal immunization in reducing the risk of disease in infants is well recognized, and currently, antenatal immunization against influenza and pertussis is recommended in several countries [37]. Nevertheless, following the maternal pertussis immunization program in the United Kingdom, the vaccination of pregnant women with a combined tetanus, low-dose diphtheria, 5-component acellular pertussis, inactivated polio combination vaccine was found to impact infant immune response to diphtheria, pertussis, and CRM_197_-conjugate pneumococcal or meningococcal vaccines, but not to TT-conjugate vaccines, when compared to historical controls [14]. The possible increased risk of immune interference when vaccines containing several CRM_197_-conjugated antigens are coadministered has been previously discussed [38]. However, concomitant administration of CRM_197_-conjugate pneumococcal or meningococcal vaccines in infants was shown not to affect immune responses to either of the vaccines [39]. In our study, following maternal immunization with the investigational GBS vaccine, the percentages of infants achieving seroprotective antibody concentrations against diphtheria were comparable to those in the placebo group, in line with previous reports [17]. The same was observed for immune responses against each serotype of the CRM_197_-conjugate PCV13 except serotype 14, which were lower in the 2 groups with the highest GBS vaccine dose. A possible explanation for this finding is that the capsular epitope of GBS serotype III included in the vaccine induced antibodies that cross-reacted with *Streptococcus pneumoniae* serotype 14 vaccine polysaccharide, due to the very similar structure of the 2 capsular polysaccharides [40]. The impact of the GBS antibodies against serotype III on the infants’ immune response to PCV13 is in line with results from a study assessing the kinetics of antibody concentrations after maternal vaccination with a pneumococcal polysaccharide vaccine, which showed that the presence of maternal antibodies reduced the immune responses to several serotypes, including serotype 14 following immunization of infants [41].

Of note, this is also the first study reporting immune responses following PCV13 administration as a 2 + 1 vaccination schedule with an early booster given at 9 months of age. The proportions of infants with antibody concentrations ≥0.35 μg/mL against each vaccine pneumococcal serotype after the primary vaccination were similar to those previously reported after the administration of 2 primary doses of PCV13 at either 2 and 4 months or 3 and 5 months of age [42]. Furthermore, following the administration of the booster PCV13 dose at 9 months, we observed robust increases in antibody GMCs for almost all vaccine serotypes, in line with results in infants primed with 2 doses who received a booster dose at 11–12 months of age [42]. These results suggest that the PCV13 booster dose can be given from 9 months of age.

The study has several limitations. The relatively low number of infants in each group could have impacted the estimation of significant differences between GBS vaccine dosage groups in terms of antibody GMCs. The results of exploratory analyses should be interpreted with caution. As no correlate of protection for infant sera has been established, evaluating antibody functional activity by opsonophagocytic activity assays could provide a more thorough comparison between studies. Assessing diphtheria and pneumococcal antibody levels at birth might have been useful to interpret immune responses to vaccination within the first months of life. However, the randomization process should have ensured comparable baseline levels across all groups, and in addition, birth levels are less relevant when evaluating immune responses at 10 months of age.

In summary, for all vaccine dosages, transferred GBS-specific antibodies continued to be detected in infants throughout the period of infant vulnerability, suggesting that maternal immunization could be used to prevent GBS invasive disease in the first months of life. Vaccination of the mothers with any of the investigational GBS CRM_197_-conjugate vaccine dosages did not seem to have a clinically meaningful impact on immune responses to diphtheria toxoid and PCV13 in infants.

## Supplementary Data

Supplementary materials are available at *Clinical Infectious Diseases* online. Consisting of data provided by the authors to benefit the reader, the posted materials are not copyedited and are the sole responsibility of the authors, so questions or comments should be addressed to the corresponding author.

## Supplementary Material

SupplementaryMaterial

## Notes

***Author contributions.*** F. W., K. S., L. J., M. O., S. A. M., and V. N. designed the study. A. K., A. S.-t. M., C. L. C., F. W., K. S., L. J., M. O., N. G., P. M. D., S. A. M., and V. N. acquired the data. A. S.-t. M., C. L. C., F. W., K. S., L. J., M. O., P. M. D., S. B., S. A. M., and V. N. analyzed the data. A. K., C. L. C., F. W., L. J., N. G., P. M. D., and S. A. M. contributed to the conduct of the study. All authors reviewed and revised the manuscript, and approved the final manuscript as submitted.

***Acknowledgments.*** The authors thank all the participants, study nurses, and staff members who contributed to this study; Petronela M. Petrar (XPE Pharma & Science) for medical writing support; and Stéphanie Deroo (XPE Pharma & Science c/o GSK) for manuscript development and editorial support. The authors also thank Lisa Bedell for assistance with statistical analysis, Pietro Forte for clinical assistance, and Jennifer Engelmoer for providing writing and editorial assistance in the preparation of this manuscript (all Novartis Vaccines Division).

***Financial support.*** The study was supported by the Novartis Vaccines Division; on 2 March 2015, Novartis’ noninfluenza vaccines business was acquired by the GSK group of companies. GlaxoSmithKline Biologicals SA took responsibility for all costs associated with the development and publishing of the present manuscript.

***Potential conflicts of interest.*** F. W., A. S.-t. M., P. M. D., and K. S. were employees of the Novartis Vaccines Division at the time of the study. V. N. is an employee of the Novartis Vaccines Division. K. S. and V. N. hold stock/stock options of Novartis Vaccines. M. O. is an employee of the GSK group of companies. S. B. is a consultant statistician for the GSK group of companies GBS project. C. L. C. declares that her institution has received grants from Novartis Vaccines and the GSK group of companies for the conduct of clinical trials. S. A. M. declares that his institution has received grants from the GSK group of companies, Pfizer, Minervax, and the Bill & Melinda Gates Foundation, and also declares having received consulting fees from Pfizer and payment for the development of educational presentations from Medscape. All other authors report no potential conflicts. All authors have submitted the ICMJE Form for Disclosure of Potential Conflicts of Interest. Conflicts that the editors consider relevant to the content of the manuscript have been disclosed.
