# Supplementary material for: Antibody Kinetics and Response to Routine Vaccinations in Infants Born to Women Who Received an Investigational Trivalent Group B Streptococcus Polysaccharide CRM197-Conjugate Vaccine During Pregnancy
Source: Clin Infect Dis. 2017 Sep 23;65(11):1897–904. doi: 10.1093/cid/cix666 (PMC5848233; doi:10.1093/cid/cix666)
Supplement: SupplementaryMaterial [file cix666_suppl_supplementarymaterial.docx]

# Supplementary Material

## Antibody kinetics and response to routine vaccinations in infants born to women who received an investigational trivalent Group B Streptococcus polysaccharide CRM_197_-conjugate vaccine during pregnancy

Shabir A. Madhi et al.

Antibody half-life measurement

The infant GBS serotype-specific antibody half-life was computed from the slope of the descending log-linear curve of concentration by time measured in days. Subjects with antibody concentrations below the LLQ at all timepoints were excluded from the analysis. Modelling was performed on subjects that had antibody levels reported at each timepoint and only subjects with decreasing concentrations were included in the analysis (approximately 79-85% of subjects across GBS serotypes Ia, Ib and III). This represents a limitation to the half-life antibody measurements as it may bias the estimated values.

Exploratory analyses

Exploratory analyses of the persistence of immune responses to GBS serotypes were carried out in subsets of infants classified by gestational age at vaccination (28 to <30 weeks, 30 to <32 weeks, 32 to <34 weeks, and ≥34 weeks) and in subsets of infants born to mothers with pre-vaccination antibody GMCs below the LLQ. Premature children (6–9% across groups, [1]) were not excluded from these analyses.

1. Madhi SA, Cutland CL, Jose L, et al. Safety and immunogenicity of an investigational maternal trivalent group B Streptococcus vaccine in healthy women and their infants: a randomised phase 1b/2 trial. Lancet Infect Dis 2016; 16(8): 923-34.

**Table S1.** GBS serotype-specific antibody GMCs in infants, by gestational age at vaccination, by timepoint (per-protocol set)

|  | **GMCs (95% CI)** | | | | | | | | | | | | | | | | | | | | | | | |  |
| --- | --- | --- | --- | --- | --- | --- | --- | --- | --- | --- | --- | --- | --- | --- | --- | --- | --- | --- | --- | --- | --- | --- | --- | --- | --- |
|  | **Placebo** | | | |  | **GBS 0.5 μg** | | | | | |  | **GBS 2.5 μg** | | | | | |  | **GBS 5.0 μg** | | | | | |
|  | **28–<30w** | **30–<32w** | **32–<34w** | **≥34w^a^** |  | | **28–<30w** | **30–<32w** | **32–<34w** | **≥34w^a^** |  | | | **28–<30w** | **30–<32w** | **32–<34w** | **≥34w^a^** |  | | | **28–<30w w** | **30–<32w** | **32–<34w** | **≥34w^a^** |  |
| **Serotype Ia** | | | | | | | | | | | | | | | | | | | | | | | | |  |
| N | 14 | 22 | 16 | 1 |  | | 17 | 17 | 15 | 4 |  | | | 16 | 14 | 21 | 1 |  | | | 18 | 13 | 9 | 1 |  |
| Birth | 0.44 | 0.45 | 0.43 | 3.9 |  | | 5.49 | 6.98 | 8.81 | 5.26 |  | | | 9.09 | 11.0 | 9.61 | 2.90 |  | | | 12.0 | 9.44 | 5.37 | 44.0 |  |
|  | 0.19-1.00 | 0.22-0.92 | 0.16-1.14 | - |  | | 2.11-14.0 | 2.12-23.0 | 2.93-26.0 | 0.70-40.0 |  | | | 3.39-24.0 | 2.89-40.0 | 3.79-24.0 | - |  | | | 4.72-30.0 | 2.41-37.0 | 1.30-22.0 | - |  |
| D43 | 0.32 | 0.32 | 0.30 | 1.7 |  | | 3.18 | 3.57 | 4.06 | 2.28 |  | | | 4.6 | 4.21 | 3.91 | 2.60 |  | | | 4.97 | 5.40 | 2.11 | 10.0 |  |
|  | 0.17-0.63 | 0.18-0.57 | 0.13-0.70 | - |  | | 1.28-7.93 | 1.16-11.0 | 1.39-12.0 | 0.34-15.0 |  | | | 1.79-12.0 | 1.22-15.0 | 1.58-9.68 | - |  | | | 2.05-12.0 | 1.50-20.0 | 0.53-8.45 | - |  |
| D91 | 0.43 | 0.45 | 0.34 | 0.9 |  | | 2.01 | 2.77 | 1.86 | 1.41 |  | | | 2.72 | 3.06 | 2.26 | 2.40 |  | | | 2.70 | 2.29 | 1.68 | 4.40 |  |
|  | 0.23-0.81 | 0.26-0.78 | 0.19-0.61 | - |  | | 0.81-4.95 | 1.16-6.63 | 0.78-4.43 | 0.13-15.0 |  | | | 1.07-6.90 | 1.17-7.99 | 1.08-4.71 | - |  | | | 1.12-6.49 | 0.84-6.21 | 0.55-5.16 | - |  |
| **Serotype Ib** | | | | | | | | | | | | | | | | | | | | | | | | |  |
| N | 11 | 18 | 15 | 1 |  | | 18 | 15 | 15 | 4 |  | | | 16 | 13 | 18 | 1 |  | | | 17 | 13 | 10 | 1 |  |
| Birth | 0.16 | 0.18 | 0.20 | 0.10 |  | | 1.38 | 1.12 | 1.38 | 1.11 |  | | | 1.88 | 2.19 | 2.24 | 7.80 |  | | | 2.33 | 1.49 | 0.45 | 0.50 |  |
|  | 0.06-0.47 | 0.08-0.40 | 0.06-0.61 | - |  | | 0.43-4.41 | 0.31-4.12 | 0.44-4.39 | 0.02-73.0 |  | | | 0.55-6.48 | 0.54-8.85 | 0.78-6.41 | - |  | | | 0.70-7.73 | 0.37-6.02 | 0.11-1.84 | - |  |
| D43 | 0.24 | 0.16 | 0.12 | 0.30 |  | | 0.95 | 0.55 | 1.00 | 0.83 |  | | | 1.43 | 1.40 | 0.92 | 3.70 |  | | | 1.10 | 0.63 | 0.32 | 0.30 |  |
|  | 0.10-0.55 | 0.08-0.32 | 0.04-0.36 | - |  | | 0.35-2.64 | 0.17-1.73 | 0.38-2.66 | 0.06-12.0 |  | | | 0.49-4.20 | 0.41-4.84 | 0.38-2.25 | - |  | | | 0.39-3.12 | 0.18-2.18 | 0.10-1.07 | - |  |
| D91 | 0.48 | 0.46 | 0.48 | 0.80 |  | | 1.57 | 0.66 | 0.95 | 1.15 |  | | | 1.60 | 1.24 | 0.80 | 2.20 |  | | | 1.37 | 0.79 | 0.58 | 0.10 |  |
|  | 0.23-1.00 | 0.27-0.79 | 0.22-1.01 | - |  | | 0.75-3.30 | 0.26-1.67 | 0.44-2.07 | 0.14-9.16 |  | | | 0.73-3.50 | 0.46-3.37 | 0.39-1.62 | - |  | | | 0.64-2.93 | 0.29-2.16 | 0.22-1.50 | - |  |
| **Serotype III** | | | | | | | | | | | | | | | | | | | | | | | | |  |
| N | 9 | 14 | 10 | 0 |  | | 10 | 10 | 9 | 2 |  | | | 12 | 12 | 10 | 1 |  | | | 13 | 5 | 6 | 1 |  |
| Birth | 0.31 | 0.19 | 0.46 | - |  | | 3.36 | 1.84 | 1.36 | 3.00 |  | | | 2.29 | 2.83 | 3.41 | 9.10 |  | | | 1.36 | 12.0 | 2.13 | 2.60 |  |
|  | 0.13-0.73 | 0.11-0.33 | 0.08-2.62 | - |  | | 1.16-9.73 | 0.50-6.82 | 0.26-7.24 | - |  | | | 0.87-6.06 | 0.86-9.36 | 0.70-17.0 | - |  | | | 0.53-3.44 | 1.91-78.0 | 0.28-16.0 | - |  |
| D43 | 0.23 | 0.10 | 0.25 | - |  | | 2.14 | 0.80 | 0.97 | 0.83 |  | | | 0.98 | 1.12 | 1.06 | 2.50 |  | | | 0.58 | 3.81 | 0.93 | 0.50 |  |
|  | 0.11-0.48 | 0.07-0.16 | 0.05-1.36 | - |  | | 0.87-5.25 | 0.23-2.78 | 0.22-4.19 | - |  | | | 0.43-2.22 | 0.36-3.49 | 0.26-4.27 | - |  | | | 0.26-1.27 | 0.66-22.0 | 0.15-5.61 | - |  |
| D91 | 0.26 | 0.17 | 0.37 | - |  | | 1.20 | 0.61 | 0.56 | 0.53 |  | | | 0.49 | 0.95 | 0.67 | 1.40 |  | | | 0.45 | 1.50 | 1.14 | 0.40 |  |
|  | 0.12-0.56 | 0.10-0.31 | 0.12-1.14 | - |  | | 0.57-2.55 | 0.25-1.44 | 0.17-1.82 | - |  | | | 0.25-0.96 | 0.43-2.11 | 0.22-2.05 | - |  | | | 0.23-0.86 | 0.44-5.13 | 0.27-4.85 | - |  |

CI, confidence interval; D, day; GMC, geometric mean concentration; N, number of infants with available results in each group; w, weeks of gestational age at vaccination.

^a^ Few pregnant women were vaccinated at ≥34 weeks of gestation and therefore GMCs for this group should be interpreted with caution and assessed for their clinical relevance.
